# *Myrianthus arboreus* P. Beauv (Cecropiaceae) Extracts Accelerates Sexual Maturation, and Increases Fertility Index and Gestational Rate in Female Wistar Rats

**DOI:** 10.3390/medicines5030073

**Published:** 2018-07-07

**Authors:** Charline Florence Awounfack, Marie Alfrede Mvondo, Stéphane Zingue, Sylvin Benjamin Ateba, Sefirin Djiogue, Rosette Megnekou, Derek Tantoh Ndinteh, Dieudonné Njamen

**Affiliations:** 1 Laboratory of Animal Physiology, Department of Animal Biology and Physiology, Faculty of Science, University of Yaounde I, P.O. Box 812, Yaounde, Cameroon; c.awounfack@yahoo.fr (C.F.A.); s.benjaminateba@gmail.com (S.B.A.); sefirindjiogue@gmail.com (S.D.); megnekor@yahoo.fr (R.M.); 2 Laboratory of Animal Physiology and Phytopharmacology, Department of Animal Biology, Faculty of Science, University of Dschang, P.O. Box 67, Dschang, Cameroon; Mvondo.mariealfrede@yahoo.com; 3 Laboratory of Physiology, Department of Life and Earth Sciences, Higher Teachers’ Training College, University of Maroua, P.O. Box 55, Maroua, Cameroon; stephanezingue@gmail.com; 4 Department of applied Chemistry, Faculty of Science, University of Johannesburg, P.O. Box 17011, Gauteng, South Africa; dndinteh@uj.ac.za

**Keywords:** female infertility, folliculogenesis, gestational rate, gonadotrophins, *Myrianthus arboreus*, Wistar rats

## Abstract

**Background:** Despite the wide use of leaves of *Myrianthus arboreus* (Cecropiaceae) in several African countries including Cameroon as food and against amenorrhea and female infertility, it has never been tested for this purpose. **Methods:** Using immature female Wistar rats, the impact of *M. arboreus* on the sexual maturation parameters (vaginal opening, ovarian relative weight and follicle maturation, gonadotropins and ovarian hormones serum levels) and fertility index has been evaluated through a 30-day oral administration of aqueous and methanol extracts of leaves at the doses of 20, 110 and 200 g/kg/day. **Results:** Aqueous extract increased the ovarian relative weight (*p* < 0.001), progesterone (*p* < 0.001) and gonadotropins (*p* < 0.001) serum levels, and induced the maturation of ovarian follicles. The methanol extract additionally induced an early vaginal opening (*p* < 0.001), uterine growth (*p* < 0.01) and increased estradiol (*p* < 0.001) serum levels. The fertility index generally increased following treatments, while the gestation rate remained almost unaffected except at the highest tested dose of *M. arboreus extracts* where lowest values were observed. **Conclusion:** Globally, *M. arboreus* induced an early puberty onset and an increased fertility rate validating at least in part its traditional use for female infertility.

## 1. Introduction

Infertility, or the failure of getting pregnant after one year of regular and unprotected intercourse [1], is sometime devastating for couples as it is very often associated with psychological distress, low self-esteem, abuse, divorce, loss of respect from extended family and even the annulment of rights to burial grounds [1,2,3,4,5,6,7,8]. It affects 10–15% of the world population at the reproductive age [9,10], while the rates in parts of developing world could exceed 30% because of the higher burden of sexually transmitted infections/diseases, the unsafe abortion and postpartum pelvic infection [7,11,12,13,14].

Female and male factors alone account for 35% and 30% of infertility, respectively, while 20% is attributed to both male and female, and 15% to unknown factors [15]. The causes of infertility in women include many types of problems, such as hyperprolactinemia and ovulatory, uterine or outflow tract and tubal factors [16]. Ovulatory dysfunction is the most common cause (25 to 50%) [17,18] and is mainly due to hormonal problems related to the dysfunction/malfunction of the hypothalamic-pituitary-ovarian axis. Beyond the efficacy, the conventional ovulation-induction therapies (exogenous gonadotropins or clomiphene citrate) [19,20] have been associated with side effects, including the ovarian hyperstimulation syndrome, myocardial infarction and ovarian cancer [20,21,22,23]. In line with this, people increasingly seek alternatives such as plant-derived medicines thought to be safer and playing an important role in health care systems, especially in the developing world where up to 80% of the population rely on traditional medicine for their primary healthcare.

*Myrianthus arboreus* P. Beauv (Cecropiaceae) is a tree widely distributed in West, Central and East Africa where its young leaves are widely used as food/vegetables [24,25,26,27] and against various sicknesses and diseases [25,28,29,30]. Aqueous extracts of leaves are traditionally used in Cameroon against amenorrhea, female infertility (primary and secondary) and to improve lactation [25,31]. Studies published over the last decade revealed antioxidant [26,32,33], antibacterial [34], anti-infective and wound healing properties [28], hypoglycaemic and antihyperlipidaemic activities [35], and antidiabetic potential through cell-based bioassays [36] of this plant. In our previous study (safety evaluation), the oral administration of an aqueous extract of *M. arboreus* for 28 days in young adult (7–8 weeks old) Wistar rats increased ovarian and uterine wet weights, and the number of Graafian follicles and corpora lutea [37] suggesting the capacity to induce the production of sexual hormones, folliculogenesis and ovulation, and increase fertility index. In line with this, the present study was carried out to evaluate the effects of extracts of *M. arboreus* on sexual maturation and some parameters of the fertility using immature female Wistar rats, a widely used and suitable model (in vivo) for this purpose [38,39,40]. Immature female rats are used because of their intact and non-functional hypothalamic-pituitary-ovarian axis to ensure low levels of active endogenous estrogens. In line with this, animals were also fed with a soy-free diet to minimize the occurrence of natural phytoestrogens. As endpoints of the study, the pituitary secretion of gonadotropins, ovarian folliculogenesis and hormones production, vaginal opening, uterine and vaginal growths, fertility index and gestation rate have been evaluated.

## 2. Materials and Methods

### 2.1. Plant Collection, Identification and Extracts

Young fresh leaves of *Myrianthus arboreus* were collected in June 2013 from the cliff forest of Dschang (West Region, Cameroon). The plant was identified and authenticated by a botanist, Mr. Victor Nana, by comparing a botanical sample with the voucher specimen registered at the Cameroon National Herbarium under the number 34045/HNC. Leaves were sorted to remove any contaminants, dead matter and washed. They were air-dried for a week and the dried leaves were ground. In this study, two methods of extraction were used. Following the traditional recommendation, 250 g of this powder was macerated in 5 L of water for 12 h (2 times) at room temperature and triturated. After filtration with Wattman paper Nr. 4, the filtrate was freeze-dried and 13.94 g of extract (AE) were obtained. Another 500 g of the powder Secs was macerated in 5 L of 95% methanol for 72 h (3 times). The filtrate was evaporated using a rotary evaporator (337 mbar at 40 °C) and 26 g of dried methanol extract (ME) were obtained (yield of extraction 5.2%).

In this study, the doses of 20, 110 and 200 mg/kg/day were extrapolated according to the recommendations of traditional healers dealing with female infertility as previously described [37].

### 2.2. Animals

Twenty-one-day-old immature female Wistar rats (average weight of 30 g) were used. They were bred in the animal house of the Laboratory of Animal Physiology, University of Yaounde I (Yaounde, Cameroon) under natural conditions (cycles of ~12 h of light and dark). They had free access to diet and drinking water ad libitum. Animal housing and experiments were carried out following the guidelines of the Institutional Ethics Committee of the Cameroon Ministry of Scientific Research and Innovation (Reg. no. FWA-IRD 0001954, 04/09/2006), which has adopted the guidelines established by the European Union on Animal Care (CEE Council 86/609).

### 2.3. Experimental Protocol

Eighty-four animals were randomly allocated to the following treatment groups (*n* = 12): control (distilled water), AE (20, 110 and 200 mg/kg BW/d) and MeOH (20, 110 and 200 mg/kg BW/d). All animals received a soy-free standard diet throughout the experiment and were treated (*per os*) once daily (10:00 a.m. to 11:00 a.m.) for 30 days from the postnatal day 21. The vaginal opening (a marker for puberty onset) was daily checked until the day it occurred, and was recorded. At the end of treatments and after a 12-h overnight fasting, five animals in each group were randomly selected and sacrificed by decapitation after light anesthesia by diazepam–ketamine *i.p.* injection (10 and 50 mg/kg BW, respectively) and separated from their wet nurse. Blood samples were collected for biochemical analysis in dry tubes. The ovaries, uteri and vagina were dissected and weighed (except vagina). The left ovary and uterus from each animal, as well as the vagina, were fixed in 10% formaldehyde for histological analysis. The right ovary was homogenized in 10% sodium–phosphate buffer (pH 7.1) and the supernatant obtained following centrifugation (3000 rpm at 5 °C for 15 min) was stored at −20 °C for the tissue total cholesterol evaluation. Blood samples collected in dry tubes were also centrifuged at 3000 rpm at 5 °C for 15 min, and the serum obtained was kept at −20 °C until use. 

The remaining female rats (7/group) were crossbred with males (one sexually experienced male for two females) until the appearance of a vaginal plug or a vaginal smear containing sperm (indicators of the gestation). Vaginal plugs or vaginal smears with sperm were examined daily (8:00 a.m. to 9:00 a.m.). The mating period was extended to the nubile age of 90 days old. During this period, the first successful conception was determined either by vaginal plug or sperm in the vagina and the pregnant rats were housed individually. The litter size was also recorded at the end of the experiment. The fertility index and gestational rate were calculated as follows: fertility index = (number of pregnant/number of mated) × 100; gestation rate = (number of females with viable fetuses at birth/total number of gestational females) × 100 [38,41]. 

### 2.4. Measurement of Biochemical Parameters

Serum and homogenates of uterus and ovary were used for biochemical analyses. 

In the serum, Follicle Stimulating Hormone (FSH), Luteinizing Hormone (LH), Estradiol and Progesterone were measured in duplicate using reagent kits from Human Elisa test (Wiesbaden, Germany) while total cholesterol (TC) in serum and ovaries were measured using reagent kits from Fortress Diagnostics Limited (Antrim, UK). 

### 2.5. Histopathological Evaluation

In addition, 5 μm thick sections of paraffin embedded tissues (uterus, vagina and ovaries) were prepared and stained with hematoxylin–eosin. The photomicroscopic observation/analysis (uterine and vaginal epithelial heights, identification of the ovarian follicles) was performed using a complete set of Zeiss (Hallbermoos, Germany) equipment (microscope Axioskop 40 and the software programs MRGrab 1.0 (Carl Zeiss, Hallbermoos, Germany, 2001) and AxioVision 3.1 (Carl Zeiss, Hallbermoos, Germany, 2001) installed in a computer). Regarding the folliculogenesis, the tenth section from each ovary was selected. We considered as primary the follicles composed of oocytes surrounded with one layer of cuboidal follicular cells; secondary or preantral follicles those with more than one follicular cell layer; and antral follicles those with present antrum of follicular fluid and graafian follicles with one single large antrum of follicular fluid. Ruptured follicles with hypertrophied follicular cells and cavity filled with blood were considered as corpora lutea.

### 2.6. Statistical Analysis

Data were expressed as mean ± standard error of the mean (SEM). The percentage of vaginal opening was statistically analyzed using ANOVA one-way test followed by Bonferroni’s test. Fisher’s exact test was used for the fertility index and gestation rate and ANOVA one-way followed by Dunnett’s test for the others data. All of these tests were performed using GraphPad Prism 5.03 software (La Jolla, CA, USA, 2009). Differences were considered significant at *p* < 0.05.

## 3. Results

### 3.1. Vaginal Opening

**Figure 1:**
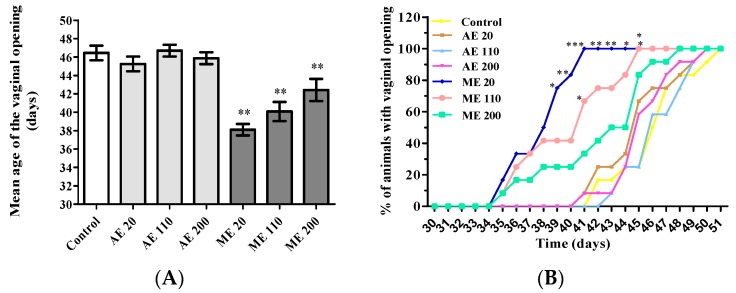
Mean age (**A**) and percentage (**B**) of animals with vaginal aperture. Control = animals receiving vehicle (distilled water, 10 mL/kg BW); AE = animals treated with the aqueous extract of *M. arboreus*; ME = Animal treated with the methanol extract of *M. arboreus*. Results are shown as the mean ± SEM.; *: *p* < 0.05; **: *p* < 0.01; ***: *p* < 0.001 in reference to control.

Treatment with the methanol extract of *M. arboreus* (ME) at the doses of 20, 110 and 200 mg/kg advanced the age of vaginal aperture by eight, six and four days, respectively, in reference to controls (Figure 1A). Concerning the cumulative percentage of vaginal aperture, a dose-dependent effect was observed with ME, the dose of 20 mg/kg being the most active (100% of vaginal aperture at the day 41). This percentage was obtained at the days 50 and 51 in the group treated aqueous extract (all the tested doses) and control animals, respectively (Figure 1B). 

### 3.2. Relative Weight of Ovaries and Uterus

**Figure 2:**
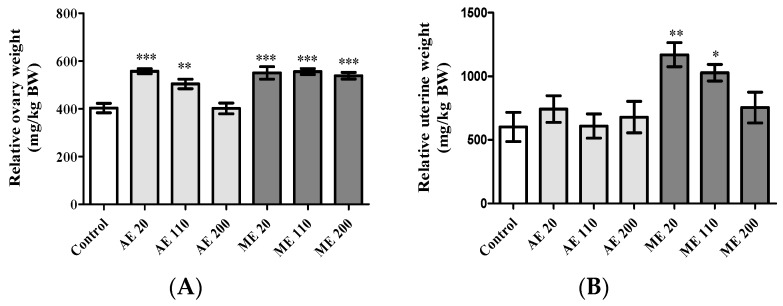
Relative weight of ovaries (**A**) and uteri (**B**) following treatments. Control = Animals receiving vehicle (distilled water, 10 mL/kg BW); AE = animals treated with the aqueous extract of *M. arboreus*; ME = animals treated with the methanol extract of *M. arboreus*. Results are shown as the mean ± SEM; *: *p* < 0.05; **: *p* < 0.01; ***: *p* < 0.001 in reference to control.

AE increased (*p* < 0.01) the ovarian relative wet weight at the doses of 20 (557.25 ± 10.69 mg/kg) and 110 (504.43 ± 19.91 mg/kg) mg/kg BW, while ME increased (*p* < 0.001) it by ~35% at all the tested doses (Figure 2A).

Concerning the uterus, only ME increased (*p* < 0.05) the relative wet weight with values of 1169.37 ± 94.47 and 1028.03 ± 65.77 mg/kg at the doses of 20 and 110 mg/kg BW, respectively, in reference to control (602.01 ± 114.41 mg/kg) (Figure 2B). 

### 3.3. Epithelial Height of Uterus and Vaginas

**Figure 3:**
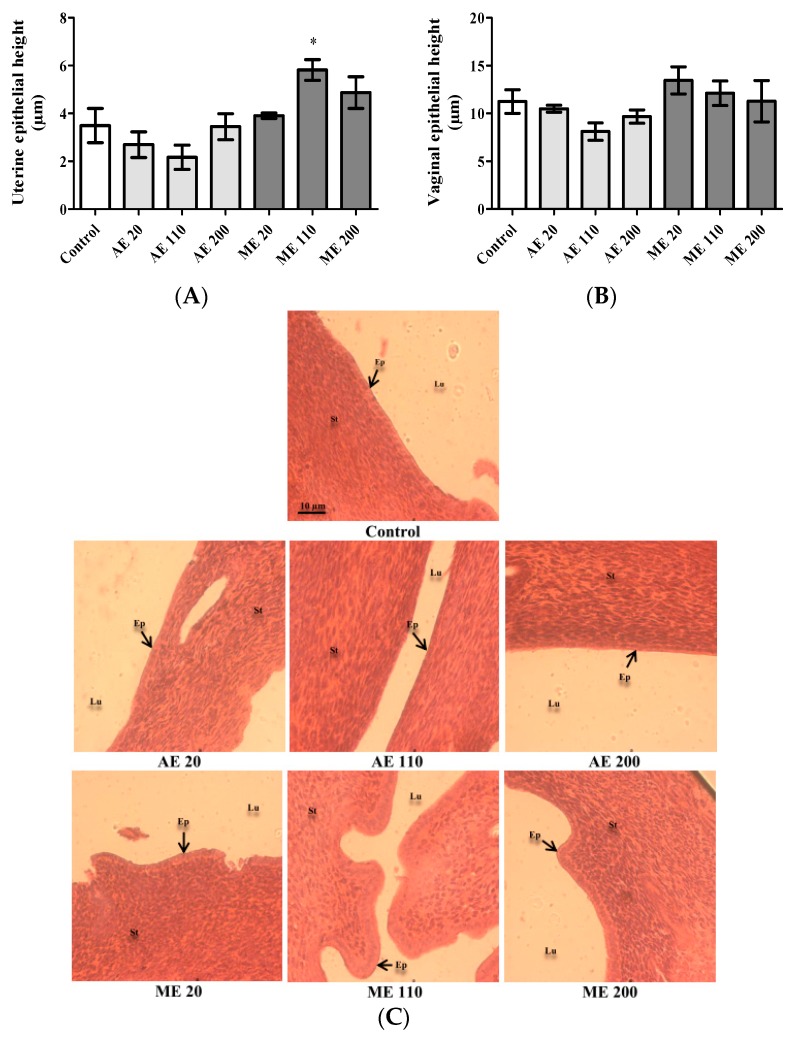
Epithelial height of uteri (**A**) and vagina (**B**) as well as microphotographs of uteri sections (**C**) following treatments. Control = Animals receiving vehicle (distilled water, 10 mL/kg BW); AE = animals treated with the aqueous extract of *M. arboreus*; ME = animals treated with the methanol extract of *M. arboreus*. Lu = lumen; Ep = epithelium; St = stroma. Results are shown as the mean ± SEM; *: *p* < 0.05 in reference to control.

After a 30-day treatment period, ME (110 mg/kg) increased the height of the uterine epithelium by 321.72% (*p* < 0.01) in reference to the value in control (3.49 ± 0.71 μm) (Figure 3A). Following treatment with extracts, no significant variation was observed in vaginal epithelial heights (Figure 3B). On the microphotographs (Figure 3C), the uteri of controls and animals treated with AE exhibited a thin layer of cuboidal cells. However, treatment with ME, especially at the dose of 110 mg/kg, was accompanied by a thickening of the epithelial layer whose cubic cells were transformed into columnar cells (pseudostratified epithelium). The stroma of controls and animals treated with AE (all tested doses) and ME (20 mg/kg) is relatively indifferentiated, while it is differentiated with slight edema at the doses of 110 and 200 mg/kg. 

### 3.4. Ovarian Follicles

**Figure 4:**
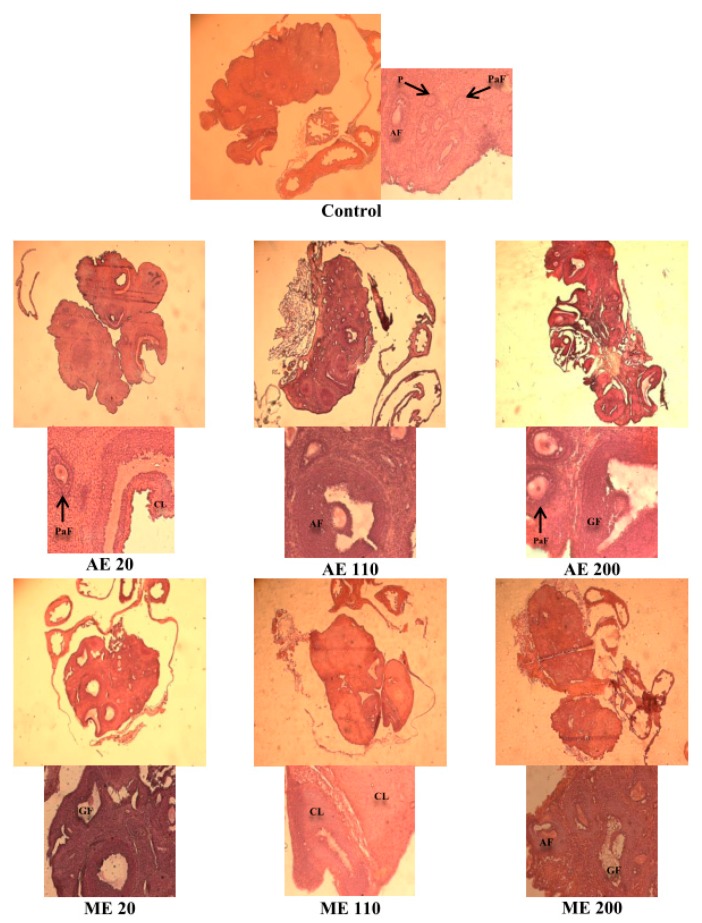
Microphotographs (25× and 200×) of hematoxylin/eosin stained sections of ovaries following a 30-day treatment with aqueous (AE) and methanol (ME) extracts of the leaves of *M. arboreus* at the doses of 20, 110 and 200 mg/kg. P = primary follicle, PF = primordial follicle, PaF = preantral follicle, AF = antral follicle, GF = Graafian follicle, CL = corpora lutea.

A 4-fold increase (*p* < 0.01) of the number of Graafian was observed with AE at the dose 200 mg/kg BW. Concerning ME, the number of antral follicles and corpora lutea increased (*p* < 0.05) by 175 and 200%, respectively, at the dose of 110 mg/kg BW (Table 1 and Figure 4).

### 3.5. Serum and Ovarian Total Cholesterol

**Figure 5:**
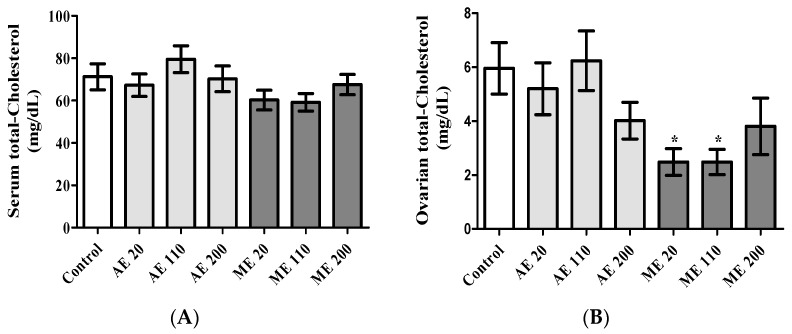
Serum (**A**) and ovarian (**B**) total cholesterol levels following treatments. Control = Animals receiving vehicle (distilled water, 10 mL/kg BW); AE = animals treated with the aqueous extract of *M. arboreus*; ME = animals treated with the methanol extract of *M. arboreus*. Results are shown as the mean ± SEM; *: *p* < 0.05 in reference to control.

Serum total cholesterol was not significantly affected following treatments (Figure 5A). In the ovaries, ME at the doses of 20 and 110 mg/kg decreased (*p* < 0.05) total cholesterol by 58.33% (Figure 5B). 

### 3.6. Hormone Levels

**Figure 6:**
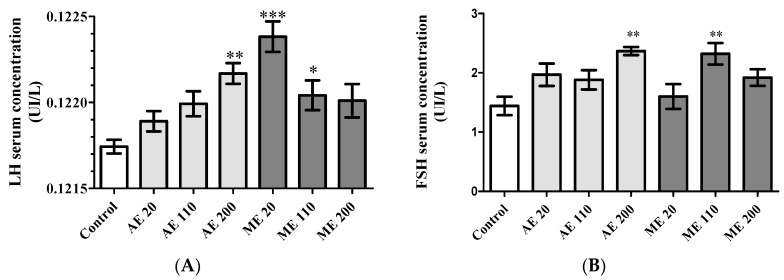

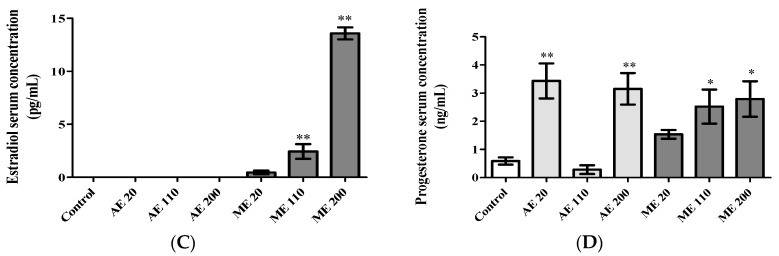
Serum levels of Luteinizing Hormone (LH) (**A**); Follicle Stimulating Hormone (FSH) (**B**) estradiol (**C**) and progesterone (**D**). Control = Animals receiving vehicle (distilled water, 10 mL/kg BW); AE = animals treated with the aqueous extract of *M. arboreus*; ME = animals treated with the methanol extract of *M. arboreus*. Results are shown as the mean ± SEM; *: *p* < 0.05; **: *p* < 0.01; ***: *p* < 0.001 in reference to control.

Treatment with AE at the dose of 200 mg/kg significantly increased (0.34%, *p* < 0.01) the serum levels of LH (Figure 6A), while ME induced it at 20 mg/kg BW (0.52%, *p* < 0.001) and 110 mg/kg BW (0.24%, *p* < 0.05). FSH serum levels generally increased following treatments. AE significantly increased it at the only dose of 200 mg/kg BW (64.11%, *p* < 0.01), while ME increased it (60.99%, *p* < 0.01) at the dose of 110 mg/kg BW in reference to control (1.44 ± 0.15 UI/L) (Figure 6B). 

As depicted in Figure 6C, ME induced a dose-dependent increase (*p* < 0.001) of estradiol serum levels with significant variations at 110 (2.43 ± 0.69 pg/mL) and 200 (13.57 ± 0.56 pg/mL) mg/kg BW. 

Regarding serum progesterone levels, a U-shaped response was obtained with AE (Figure 6D)*.* It increased serum progesterone levels at 20 mg/kg BW (481.42%, *p* < 0.01) and 200 mg/kg BW (433.82%, *p* < 0.01), while decreasing it at 110 mg/kg BW (52.43% induction) (Figure 6D). ME in contrast induced a dose-dependent increase of the serum progesterone levels with significant values at 110 mg/kg BW (327.05%, *p* < 0.05) and at 200 mg/kg BW (372.51%, *p* < 0.05). 

### 3.7. Effects of M. arboreus Extracts on Fertility and Gestation

Fertility index generally increased following treatments (Table 2). This index exceeded 50% in animals receiving AE at 110 mg/kg and ME at 20 mg/kg and 110 mg/kg. Regarding the gestation rate, the lowest values of 50 and 33.33% were obtained with AE and ME, respectively, at the highest dose of 200 mg/kg (Table 2).

## 4. Discussion

The present study was designed to evaluate the effects of the aqueous and methanol leaves extracts of *Myrianthus arboreus* on some biological parameters of reproduction, especially on physiological parameters of the pubertal onset (age of vaginal opening), morphological changes of reproductive organs (ovaries, vagina and uteri), some gonadotrophic and steroidal hormones, and some fertility parameters. 

Sexual maturation is attained during puberty of which onset requires changes in the sensitivity, activity and function of the hypothalamic-pituitary-gonadal axis. Without these changes, the process of sexual development remains incomplete and a functional reproductive system not fully developed. In other words, sexual maturation or puberty is a prerequisite to being fertile. Vaginal opening is known to be the initial and external sign of estrogenic rise that accompanies the onset of puberty [42,43]. Our results show that, in contrast to AE, the 30-day treatment with ME at the doses of 20, 110 and 200 mg/kg BW advanced vaginal opening by eight, six and four days, respectively, in reference to the control group (postnatal day 46.44 ± 0.78). These results strongly suggest the ovarian secretion of estrogen or the presence of estrogen-like compounds (phytoestrogens) in the methanol extract. Interestingly, the prominent effect of ME was observed with dose of 20 mg/kg. Compared to the aqueous extract that did not advance the vaginal opening, the methanol extract of *M. arboreus* may contain active secondary metabolites missing or present in low relative abundance in the aqueous extract to induce the advance the puberty onset. Moreover, it is known that, compared to water, methanol extraction has a much higher yield of extraction of total phenolics as well as total flavonoids, which are known to exhibit estrogenic activity [44].

Besides vaginal opening, reproductive maturation is associated with the activation of the hypothalamic-pituitary-ovarian axis and an appropriate quantity along with the sequence of gonadotropins (follicle stimulating hormone (FSH) and luteinizing hormone (LH), and of the two ovarian hormones (estrogen and progesterone) responsible for the development and function of estrogen sensitive organs including breast, uterus, and vagina. On this basis, the relative weight of uterus and ovaries or the uterine and vaginal epithelial heights constitute crucial benchmarks to evaluate the impact of chemicals on the female reproductive tract/system [45,46]. While uterine growth (water imbibition and/or cell proliferation) is stimulated by estrogens or estrogen-like compounds [47,48], ovarian development and function required both gonadotrophic (FSH and LH) and estrogenic compounds [49,50,51]. Both AE (20 and 110 mg/kg) and ME (at the all tested doses) induced ovarian growth while only ME at the doses of 20 and 110 mg/kg increased the uterine relative weight. The uterus is considered as an index of estrogen secretion or estrogen-like compounds effect as it constitutes along with vagina the estrogen primary target organs. Therefore, our results suggest that, contrary to the methanol extract, the ovarian growth induced by the aqueous extract is not enough to induce estrogen secretion. On the other hand, during puberty, the ovarian maturation/growth is mainly attributed to the presence of more tertiary/antral and Graafian follicles [51]. Compared to the control, AE increased the number of Graafian follicles by 150% and 400% at the doses of 20 and 200 mg/kg BW, respectively, while antral follicles were not affected except the decreased number observed at 110 mg/kg BW. However, treatment with ME increased the number of antral and Graafian follicles at all the tested doses in reference to the control, suggesting a more sustained ovarian maturation than that observed with the aqueous extract. 

Steroid hormones are derivatives of cholesterol. Therefore, decreased levels of ovarian cholesterol, associated with elevated levels of estradiol and progesterone serum concentrations, suggest a sustained increase in steroidogenesis [52]. Slight (not significant) decreases of ovarian cholesterol were observed with AE at 20 and 200 mg/kg, while ME decreased it by 58.31, 58.31 and 36.13% at the doses of 20, 110 and 200 mg/kg, respectively. The production of estradiol may be stimulated by elevated levels of FSH and LH, as these gonadotropins are known to stimulate ovarian follicle growth and the production of estrogens [53]. However, despite the increase of FSH and LH observed with AE, especially at the dose of 200 mg/kg, estradiol was not detected in the serum of these animals. The underlying mechanism of such results is not known. It is possible that the basal levels of estradiol produced during puberty were not yet reached and the low amounts produced were directly used in situ to enhance the effect of FSH on follicle growth and maturation. However, this low and non-detectable circulating level of estradiol corroborates or accounts for the lack of uterine growth observed, and, therefore, confirms the uncompleted aspect of the reproductive maturation in these animals. During the second phase of the ovarian cycle, and in response to LH, corpora lutea produces progesterone, which, in turn, inhibits LH release by the anterior pituitary in a feedback mechanism action [50,54]. This may explain at least in part why increased serum progesterone levels in animals treated with ME were inversely correlated with LH serum concentrations. However, treatment with AE at the doses of 20, 100 and 200 mg/kg induced a linear dose–response effect in the LH levels, while progesterone levels presented a U-shaped dose response or ‘hormesis’-type response (higher effect with the first and last doses). Such response is not yet fully understood [55,56,57]. However, low progesterone levels were in line with fewer corpora lutea number observed in the ovaries of animals receiving AE at the dose of 110 mg/kg. This number was similar to that observed in controls, hence progesterone levels were similar. These results suggest that, in these groups, animals were in a phase of the estrus cycle during which estradiol levels are low (e.g., early follicular phase). This hypothesis is supported by results on ovarian follicles showing a low number of antral and graffian follicles, which are known to produce an important amount of estrogens [53]. On the other hand, the progesterone levels obtained with AE at the dose of 20 and 200 mg/kg were globally higher than that obtained with ME, while the number of corpora lutea were equal or higher with ME. These results suggest the presence of developing/mature corpora lutea (characterized by the production of huge amounts of progesterone) in animals treated with aqueous extract and of regressing corpora lutea (associated progressive withdrawal of progesterone) in animals treated with ME. Globally, at the day of sacrifice, animals treated with methanol extract of *M. arboreus* displayed all or almost all of their sexual maturation, while a very complex pattern, especially with ovarian steroids, was observed with the aqueous extract. 

The fertility test showed that both extracts of *M. arboreus* of the all tested doses had an increased fertility index. After treating with AE at a dose of 100 mg/kg and with MEE at 20 and 100 mg/kg, more than 50% of female rats were pregnant. In these respective groups, 100% of gestational female rats had viable and healthy fetuses at birth, suggesting that, at these doses, *M. arboreus* induced hormonal changes and modifications in reproductive organs (ovaries and uterus) allowed implantation and development of embryos. However, both extracts reduced fertility index and gestational rate at the higher dose of 200 mg/kg. Implantation, which occurs 4–8 days after fertilization in human and rodents, is critical for the development/growth of the embryo [58]. It requires a proper or adequate priming of the uterus by estrogen and progesterone, indicating that any disturbance of this hormonal equilibrium would impede it and cause infertility [59]. Therefore, each treatment would have activated the hypothalamic-pituitary-ovarian axis in immature female rats for hormone release and then induced uterine endometrial changes indispensable for the implantation and development of the blastocyst [60]. The increase in gestational rate (ratio between the number of female rats with viable fetuses at birth and the number of gestational female rats) [38] may rest on a sustained uterotrophic effect as observed by Watcho et al. [61]. Although at the dose of 200 mg/kg, both extracts displayed a higher fertility index compared to control, this higher dose has been associated with lower gestational rates with values of 50 and 33.33% in animals receiving aqueous and methanol extract, respectively. These results indicate that, among the gestational females, only 50% and ~66% had viable fetuses at birth. Therefore, the higher doses of *M. arboreus* might be associated with an increased number of miscarriages and/or stillborn. Moreover, based on the dose used in humans against female infertility and extrapolated from the macerate of the traditional healer, i.e., 20 mg/kg [37], the equivalent dose in rats is estimated to 120 mg/kg BW using allometric calculations [62], very close to 110 mg/kg BW that presented the best profile. However, the high dose of 200 mg/kg BW in rats has an equivalent dose in humans of 32 mg/kg BW, suggesting that *M. arboreus* at such higher doses might be lethal for embryos or fetuses. Therefore, these data provide a scientific background that the dose used by the traditional healers (in Dschang) might be suitable against infertility and strongly recommended them to avoid higher doses—for instance, 0.6-fold higher. Higher fertility index and gestation rate were obtained with ME compared to AE, which is popular to use. Accordingly, to have the maximum therapeutic effects of *M. arboreus* on female infertility, its leaves need to be macerated in an alcoholic solvent. In line with this, the raffia juice or palm wine known as “matango” that constitutes the most used alcoholic solvent in folk medicine in Cameroon for plant extraction may be more appropriate.

## 5. Conclusions

The methanol extract of *M. arboreus* induced earlier sexual maturation than the aqueous extract. It may contain active secondary metabolites missing or present in relative low abundance in the aqueous extract. In addition, both extracts increased the fertility index and gestation rate, methanol extract being the more active. However, the higher dose of 200 mg/kg adversely affects the fertility index and gestation rate. Taken altogether, these results provide a substantial scientific background of the traditional use of *M. arboreus* in Cameroon against amenorrhea (through the advancement of sexual maturation) and for the improvement of fertility.

## Figures and Tables

**Table 1:** Number of different ovarian follicles and corpora lutea after 30 days of treatment.

Types of Follicles	Control	Aqueous extract of *M. arboreus* (mg/kg BW/d)			Methanol extract of *M. arboreus* (mg/kg BW/d)		
		AE 20	AE 110	AE 200	ME 20	ME 110	ME 200
**Primary follicles**	5.00 ± 0.83	5.40 ± 0.92	5.20 ± 0.48	6.80 ± 0.86	8.00 ± 1.14	5.80 ± 0.37	6.20 ± 0.73
**Preantral follicles**	9.00 ± 2.12	14.20 ± 3.08	17.60 ± 2.22	12.60 ± 1.28	16.00 ± 3.11	15.00 ± 2.81	15.40 ± 5.51
**Antral follicles**	1.60 ± 0.24	1.80 ± 1.11	0.80 ± 0.37	1.60 ± 0.67	3.40 ± 0.67	4.4 ± 0.74*	2.40 ± 0.50
**Graafian follicles**	0.40 ± 0.24	1.00 ± 0.31	0.60 ± 0,24	2.00 ± 0.31**	1.00 ± 0.31	1.40 ± 0.24	1.00 ± 0.31
**Corpora lutea**	0.60 ± 0.40	1.80 ± 0.86	0.50 ± 0.22	1.40 ± 0.67	1.40 ± 0.50	3.00 ± 0.89*	1.80 ± 0.20

Results are shown as the mean ± SEM (*n* = 5). *: *p* < 0.05; **: *p* < 0.01 in reference to control. AE = animals treated with the aqueous extract of *M. arboreus*; ME = animals treated with the methanol extract of *M. arboreus*.

**Table 2:** Effects of the aqueous and the methanol extracts of *M. arboreus* on fertility and gestation index.

Experimental Groups	Fertility Index (%)	Gestation Rate (%)
Control (distilled water 10 mL/kg)	14.28	100
AE 20 (aqueous extract 20 mg/kg)	28.57	100
AE 110 (aqueous extract 110 mg/kg)	57.14	100
AE 200 (aqueous extract 200 mg/kg)	28.57	50
ME 20 (methanol extract 20 mg/kg)	71.42	100
ME 110 (methanol extract 110 mg/kg)	71.42	100
ME 200 (methanol extract 200 mg/kg)	42.85	33.33

Number of rats per group (*n*) = 7.

## Abbreviations

FSH	Follicle-stimulating hormone;
GnRH	Gonadotropin-releasing hormone;
LH	Luteinizing hormone.
